# Supplementary material for: Bronchoalveolar Lavage Cytology in Severe Equine Asthma: Cytocentrifugated versus Sediment Smear Preparations
Source: Vet Sci. 2023 Aug 16;10(8):527. doi: 10.3390/vetsci10080527 (PMC10459724; doi:10.3390/vetsci10080527)
Supplement: Supplementary file 1 [file vetsci-10-00527-s001.zip › vetsci-2480682-supplementary.pdf]

Table S1. Raw data of cytocentrifugate and smear cell count results at T0.
Abbreviations: bd=below to detect

				T0					
	**Horse 1**	**1st field**	**2nd field**	**3rd field**	**4th field**	**5th field**	**Mean**	**%**	**Mucus**
	Neutrophils	52	54	57	52	48	53	56	
	Mast cells	1	0	0	0	0	0.2	0	
Cytospin	Lymphocytes	26	25	22	31	33	27	28	1
	Macrophages	14	16	14	16	15	15	16	
	Eosinophils	0	0	0	0	0	0	0	
	Neutrophils	bd	bd	bd	bd	bd	bd	bd	
	Mast cells	bd	bd	bd	bd	bd	bd	bd	
Smear	Lymphocytes	bd	bd	bd	bd	bd	bd	bd	bd
	Macrophages	bd	bd	bd	bd	bd	bd	bd	
	Eosinophils	bd	bd	bd	bd	bd	bd	bd	
	**Horse 2**	**1st field**	**2nd field**	**3rd field**	**4th field**	**5th field**	**Mean**	**%**	**Mucus**
	Neutrophils	5	12	7	12	13	13	23	
	Mast cells	0	0	0	0	0	0	0	
	Lymphocytes	24	27	22	25	23	24	56	0
Cytospin	Macrophages	15	10	7	6	8	9	21	
	Eosinophils	0	0	0	0	0	0	0	
	Neutrophils	22	42	53	31	47	39	24	
	Mast cells	0	0	0	0	0	0	0	
Smear	Lymphocytes	76	14	25	45	20	36	31	3
	Macrophages	52	43	41	24	40	40	35	
	Eosinophils	0	0	0	0	0	0	0	
	**Horse 3**	**1st field**	**2nd field**	**3rd field**	**4th field**	**5th field**	**Mean**	**%**	**Mucus**
	Neutrophils	43	51	57	53	49	51	55	
	Mast cells	0	0	0	0	0	0	0	
Cytospin	Lymphocytes	19	23	13	19	10	17	19	1
	Macrophages	25	16	26	28	25	24	26	
	Eosinophils	0	0	0	0	0	0	0	
	Neutrophils	31	82	108	105	94	84	71	
	Mast cells	0	0	0	0	0	0	0	
Smear	Lymphocytes	26	8	11	6	11	12	10	1
	Macrophages	23	21	28	21	18	22	19	
	Eosinophils	0	0	0	0	0	0	0	
	**Horse 4**	**1st field**	**2nd field**	**3rd field**	**4th field**	**5th field**	**Mean**	**%**	**Mucus**
	Neutrophils	5	6	7	5	5	6	14	
	Mast cells	0	0	0	0	0	0	0	
Cytospin	Lymphocytes	21	18	22	23	20	21	49	0
	Macrophages	18	16	11	18	17	16	37	
	Eosinophils	0	0	0	0	0	0	0	
	Neutrophils	bd	bd	bd	bd	bd	bd	bd	
	Mast cells	bd	bd	bd	bd	bd	bd	bd	
Smear	Lymphocytes	bd	bd	bd	bd	bd	bd	bd	bd
	Macrophages	bd	bd	bd	bd	bd	bd	bd	
	Eosinophils	bd	bd	bd	bd	bd	bd	bd	
	**Horse 5**	**1st field**	**2nd field**	**3rd field**	**4th field**	**5th field**	**Mean**	**%**	**Mucus**
	Neutrophils	bd	bd	bd	bd	bd	bd	bd	
	Mast cells	bd	bd	bd	bd	bd	bd	bd	
Cytospin	Lymphocytes	bd	bd	bd	bd	bd	bd	bd	bd
	Macrophages	bd	bd	bd	bd	bd	bd	bd	
	Eosinophils	bd	bd	bd	bd	bd	bd	bd	
	Neutrophils	6	3	3	0	2	3	13	
	Mast cells	0	0	0	0	0	0	0	
Smear	Lymphocytes	5	11	15	16	20	13	54	0
	Macrophages	5	9	6	15	7	8	33	
	Eosinophils	0	0	0	0	0	0	0	
	**Horse 6**	**1st field**	**2nd field**	**3rd field**	**4th field**	**5th field**	**Mean**	**%**	**Mucus**
	Neutrophils	20	33	22	19	28	24	39	
	Mast cells	0	0	0	0	0	0	0	
Cytospin	Lymphocytes	25	22	19	24	26	23	38	2
	Macrophages	14	15	21	10	11	14	23	
	Eosinophils	0	0	0	0	0	0	0	
	Neutrophils	65	44	60	40	38	49	52	
	Mast cells	0	0	0	0	0	0	0	
Smear	Lymphocytes	12	41	15	17	22	21	22	3
	Macrophages	25	31	25	23	15	24	26	
	Eosinophils	0	0	0	0	0	0	0	

Table S2. Raw data of cytocentrifugate and smear cell count results at T1.
Abbreviations: bd=below to detect

				T1					
	**Horse 1**	**1st field**	**2nd field**	**3rd field**	**4th field**	**5th field**	**Mean**	**%**	**Mucus**
Cytospin	Neutrophils	3	17	17	21	19	17	30	1
	Mast cells	0	0	0	1	0	0.2	0	
	Lymphocytes	28	27	28	32	30	29	52	
	Macrophages	5	10	13	10	11	10	18	
	Eosinophils	0	0	0	0	0	0	0	
Smear	Neutrophils	112	142	155	115	100	125	70	3
	Mast cells	0	0	0	0	0	0	0	
	Lymphocytes	35	45	42	37	37	39	22	
	Macrophages	14	18	13	15	15	15	8	
	Eosinophils	0	0	0	0	0	0	0	
	**Horse 2**	**1st field**	**2nd field**	**3rd field**	**4th field**	**5th field**	**Mean**	**%**	**Mucus**
Cytospin	Neutrophils	16	22	19	23	20	20	27	1
	Mast cells	0	0	1	0	0	0	0.2	
	Lymphocytes	38	39	41	39	35	38	52	
	Macrophages	10	16	14	18	15	15	21	
	Eosinophils	0	0	0	0	0	0	0	
Smear	Neutrophils	33	35	37	42	40	38	36	3
	Mast cells	0	0	0	1	0	0.2	0	
	Lymphocytes	36	13	16	22	20	21	20	
	Macrophages	45	50	57	40	45	47	44	
	Eosinophils	0	0	0	0	0	0	0	
	**Horse 3**	**1st field**	**2nd field**	**3rd field**	**4th field**	**5th field**	**Mean**	**%**	**Mucus**
Cytospin	Neutrophils	bd	bd	bd	bd	bd	bd	bd	bd
	Mast cells	bd	bd	bd	bd	bd	bd	bd	
	Lymphocytes	bd	bd	bd	bd	bd	bd	bd	
	Macrophages	bd	bd	bd	bd	bd	bd	bd	
	Eosinophils	bd	bd	bd	bd	bd	bd	bd	
Smear	Neutrophils	bd	bd	bd	bd	bd	bd	bd	bd
	Mast cells	bd	bd	bd	bd	bd	bd	bd	
	Lymphocytes	bd	bd	bd	bd	bd	bd	bd	
	Macrophages	bd	bd	bd	bd	bd	bd	bd	
	Eosinophils	bd	bd	bd	bd	bd	bd	bd	
	**Horse 4**	**1st field**	**2nd field**	**3rd field**	**4th field**	**5th field**	**Mean**	**%**	**Mucus**
Cytospin	Neutrophils	85	80	79	65	60	74	68	2
	Mast cells	0	0	0	0	0	0	0	
	Lymphocytes	23	20	10	7	19	16	15	
	Macrophages	18	20	17	14	25	19	17	
	Eosinophils	0	0	0	0	0	0	0	
Smear	Neutrophils	185	152	165	180	170	170	90	3
	Mast cells	0	0	0	0	0	0	0	
	Lymphocytes	17	5	5	8	7	8	4	
	Macrophages	11	14	13	10	11	12	6	
	Eosinophils	0	0	0	0	0	0	0	
	**Horse 5**	**1st field**	**2nd field**	**3rd field**	**4th field**	**5th field**	**Mean**	**%**	**Mucus**
Cytospin	Neutrophils	47	52	54	48	50	50	54	1
	Mast cells	0	0	0	0	0	0	0	
	Lymphocytes	28	33	30	27	29	29	31	
	Macrophages	16	14	13	14	15	14	15	
	Eosinophils	0	0	0	0	0	0	0	
Smear	Neutrophils	33	40	43	41	39	39	47	3
	Mast cells	0	0	0	0	0	0	0	
	Lymphocytes	36	43	39	38	37	39	46	
	Macrophages	5	6	7	5	6	6	7	
	Eosinophils	0	0	0	0	0	0	0	
	**Horse 6**	**1st field**	**2nd field**	**3rd field**	**4th field**	**5th field**	**Mean**	**%**	**Mucus**
Cytospin	Neutrophils	bd	bd	bd	bd	bd	bd	bd	bd
	Mast cells	bd	bd	bd	bd	bd	bd	bd	
	Lymphocytes	bd	bd	bd	bd	bd	bd	bd	
	Macrophages	bd	bd	bd	bd	bd	bd	bd	
	Eosinophils	bd	bd	bd	bd	bd	bd	bd	
Smear	Neutrophils	bd	bd	bd	bd	bd	bd	bd	bd
	Mast cells	bd	bd	bd	bd	bd	bd	bd	
	Lymphocytes	bd	bd	bd	bd	bd	bd	bd	
	Macrophages	bd	bd	bd	bd	bd	bd	bd	
	Eosinophils	bd	bd	bd	bd	bd	bd	bd	

Table S3. Raw data of cytocentrifugate and smear cell count results at T2.
Abbreviations: bd=below to detect

				T2					
	**Horse 1**	**1st field**	**2nd field**	**3rd field**	**4th field**	**5th field**	**Mean**	**%**	**Mucus**
Cytospin	Neutrophils	7	7	9	10	8	8	23	0
	Mast cells	0	0	0	0	0	0	0	
	Lymphocytes	18	25	21	26	22	22	63	
	Macrophages	5	7	4	5	4	5	14	
	Eosinophils	0	0	0	0	0	0	0	
Smear	Neutrophils	7	6	8	9	10	8	20	0
	Mast cells	0	0	0	0	0	0	0	
	Lymphocytes	24	27	29	28	28	27	68	
	Macrophages	3	9	4	3	4	5	12	
	Eosinophils	0	0	0	0	0	0	0	
	**Horse 2**	**1st field**	**2nd field**	**3rd field**	**4th field**	**5th field**	**Mean**	**%**	**Mucus**
Cytospin	Neutrophils	15	18	20	14	15	16	14	2
	Mast cells	0	0	0	0	1	0.2	0	
	Lymphocytes	58	60	61	57	59	59	51	
	Macrophages	41	45	42	38	40	41	35	
	Eosinophils	0	0	0	0	0	0	0	
Smear	Neutrophils	5	6	6	5	2	5	8	3
	Mast cells	0	0	0	0	0	0	0	
	Lymphocytes	29	35	36	43	26	34	56	
	Macrophages	20	19	28	25	19	22	36	
	Eosinophils	0	0	0	0	0	0	0	
	**Horse 3**	**1st field**	**2nd field**	**3rd field**	**4th field**	**5th field**	**Mean**	**%**	**Mucus**
Cytospin	Neutrophils	132	130	124	139	127	130	60	2
	Mast cells	1	0	0	0	0	0.2	0	
	Lymphocytes	40	41	37	43	42	41	19	
	Macrophages	42	39	43	49	45	44	21	
	Eosinophils	0	0	0	0	0	0	0	
Smear	Neutrophils	bd	bd	bd	bd	bd	bd	bd	bd
	Mast cells	bd	bd	bd	bd	bd	bd	bd	
	Lymphocytes	bd	bd	bd	bd	bd	bd	bd	
	Macrophages	bd	bd	bd	bd	bd	bd	bd	
	Eosinophils	bd	bd	bd	bd	bd	bd	bd	
	**Horse 4**	**1st field**	**2nd field**	**3rd field**	**4th field**	**5th field**	**Mean**	**%**	**Mucus**
Cytospin	Neutrophils	10	19	16	22	20	17	21	0
	Mast cells	0	0	0	0	0	0	0	
	Lymphocytes	43	51	53	45	49	48	58	
	Macrophages	14	17	20	19	14	17	21	
	Eosinophils	0	0	0	0	0	0	0	
Smear	Neutrophils	28	25	27	35	38	31	25	0
	Mast cells	0	0	0	0	0	0	0	
	Lymphocytes	64	62	69	71	63	66	52	
	Macrophages	32	24	30	31	28	29	23	
	Eosinophils	0	0	0	0	0	0	0	
	**Horse 5**	**1st field**	**2nd field**	**3rd field**	**4th field**	**5th field**	**Mean**	**%**	**Mucus**
Cytospin	Neutrophils	17	15	11	18	19	16	18	2
	Mast cells	0	2	0	0	0	0.4	1	
	Lymphocytes	44	42	49	39	48	44	49	
	Macrophages	28	31	31	30	28	30	32	
	Eosinophils	0	0	0	0	0	0	0	
Smear	Neutrophils	3	1	2	1	1	2	9	0
	Mast cells	0	0	1	0	0	0.2	1	
	Lymphocytes	15	9	18	15	17	15	64	
	Macrophages	7	6	6	5	8	6	26	
	Eosinophils	0	0	0	0	0	0	0	
	**Horse 6**	**1st field**	**2nd field**	**3rd field**	**4th field**	**5th field**	**Mean**	**%**	**Mucus**
Cytospin	Neutrophils	30	26	25	32	35	30	17	3
	Mast cells	0	0	0	0	0	0	0	
	Lymphocytes	102	98	90	104	92	97	56	
	Macrophages	43	51	44	58	40	47	27	
	Eosinophils	0	0	0	0	0	0	0	
Smear	Neutrophils	bd	bd	bd	bd	bd	bd	bd	bd
	Mast cells	bd	bd	bd	bd	bd	bd	bd	
	Lymphocytes	bd	bd	bd	bd	bd	bd	bd	
	Macrophages	bd	bd	bd	bd	bd	bd	bd	
	Eosinophils	bd	bd	bd	bd	bd	bd	bd	

Table S4. Raw data of cytocentrifugate and smear cell count results at T3.
Abbreviations: bd=below to detect

T3									
	**Horse 1**	**1st field**	**2nd field**	**3rd field**	**4th field**	**5th field**	**Mean**	**%**	**Mucus**
Cytospin	Neutrophils	105	110	100	94	99	102	58	3
	Mast cells	0	0	0	0	0	0	0	
	Lymphocytes	48	43	49	41	42	45	26	
	Macrophages	31	27	30	25	31	29	16	
	Eosinophils	0	0	0	0	0	0	0	
Smear	Neutrophils	83	96	84	100	92	91	81	3
	Mast cells	0	0	0	0	0	0	0	
	Lymphocytes	8	17	15	22	14	15	14	
	Macrophages	4	5	6	7	6	6	5	
	Eosinophils	0	0	0	0	0	0	0	
	**Horse 2**	**1st field**	**2nd field**	**3rd field**	**4th field**	**5th field**	**Mean**	**%**	**Mucus**
Cytospin	Neutrophils	170	176	186	201	175	182	75	2
	Mast cells	0	0	0	0	0	0	0	
	Lymphocytes	19	22	19	27	24	22	9	
	Macrophages	40	35	32	50	37	39	16	
	Eosinophils	0	0	0	0	0	0	0	
Smear	Neutrophils	207	215	200	225	230	215	85	3
	Mast cells	0	0	0	0	0	0	0	
	Lymphocytes	11	18	16	12	10	13	5	
	Macrophages	20	28	22	25	30	25	10	
	Eosinophils	0	0	0	0	0	0	0	
	**Horse 3**	**1st field**	**2nd field**	**3rd field**	**4th field**	**5th field**	**Mean**	**%**	**Mucus**
Cytospin	Neutrophils	180	165	172	183	190	178	74	2
	Mast cells	0	0	0	0	0	0	0	
	Lymphocytes	30	30	32	28	31	30	12	
	Macrophages	38	35	38	30	30	34	14	
	Eosinophils	0	0	0	0	0	0	0	
Smear	Neutrophils	130	128	110	135	112	123	76	2
	Mast cells	0	0	0	0	0	0	0	
	Lymphocytes	15	21	19	26	20	20	12	
	Macrophages	17	19	17	22	11	17	11	
	Eosinophils	2	1	3	0	0	1.2	1	
	**Horse 4**	**1st field**	**2nd field**	**3rd field**	**4th field**	**5th field**	**Mean**	**%**	**Mucus**
Cytospin	Neutrophils	23	15	24	19	18	20	36	2
	Mast cells	0	0	0	0	0	0	0	
	Lymphocytes	23	23	27	21	28	24	44	
	Macrophages	12	10	12	9	11	11	20	
	Eosinophils	0	0	0	0	0	0	0	
Smear	Neutrophils	14	26	24	22	26	22	34	0
	Mast cells	0	0	0	0	0	0	0	
	Lymphocytes	28	27	24	26	28	27	41	
	Macrophages	13	23	14	15	13	16	25	
	Eosinophils	0	0	0	0	0	0	0	
	**Horse 5**	**1st field**	**2nd field**	**3rd field**	**4th field**	**5th field**	**Mean**	**%**	**Mucus**
Cytospin	Neutrophils	18	24	22	19	22	21	27	2
	Mast cells	0	0	0	0	0	0	0	
	Lymphocytes	42	39	41	40	39	40	51	
	Macrophages	15	19	19	14	20	17	22	
	Eosinophils	0	0	0	0	0	0	0	
Smear	Neutrophils	132	148	141	165	172	152	81	3
	Mast cells	0	0	0	0	0	0	0	
	Lymphocytes	15	14	19	25	21	19	10	
	Macrophages	14	10	15	20	19	16	9	
	Eosinophils	0	0	0	0	0	0	0	
	**Horse 6**	**1st field**	**2nd field**	**3rd field**	**4th field**	**5th field**	**Mean**	**%**	**Mucus**
Cytospin	Neutrophils	108	110	105	98	100	104	58	1
	Mast cells	0	0	0	0	0	0	0	
	Lymphocytes	48	49	52	52	50	50	28	
	Macrophages	31	27	15	22	23	24	14	
	Eosinophils	0	0	0	0	0	0	0	
Smear	Neutrophils	72	105	48	72	74	74	69	2
	Mast cells	0	0	0	0	0	0	0	
	Lymphocytes	24	25	24	17	31	24	23	
	Macrophages	9	10	7	6	13	9	8	
	Eosinophils	0	0	0	0	0	0	0	

Table S5. Raw data of cytocentrifugate and smear cell count results at T4.
Abbreviations: bd=below to detect

T4									
	**Horse 1**	**1st field**	**2nd field**	**3rd field**	**4th field**	**5th field**	**Mean**	**%**	**Mucus**
Cytospin	Neutrophils	22	33	25	28	35	29	76	1
	Mast cells	0	0	0	0	0	0	0	
	Lymphocytes	9	5	12	5	6	7	19	
	Macrophages	3	1	4	1	2	2	5	
	Eosinophils	0	0	0	0	0	0	0	
Smear	Neutrophils	125	139	149	130	112	131	82	3
	Mast cells	0	0	0	0	0	0	0	
	Lymphocytes	11	17	15	19	19	16	10	
	Macrophages	7	12	9	15	16	12	8	
	Eosinophils	0	0	0	0	0	0	0	
	**Horse 2**	**1st field**	**2nd field**	**3rd field**	**4th field**	**5th field**	**Mean**	**%**	**Mucus**
Cytospin	Neutrophils	49	52	56	47	49	51	52	1
	Mast cells	0	0	0	0	0	0	0	
	Lymphocytes	27	33	39	37	32	34	34	
	Macrophages	12	18	15	14	12	14	14	
	Eosinophils	0	0	0	0	0	0	0	
Smear	Neutrophils	bd	bd	bd	bd	bd	bd	bd	bd
	Mast cells	bd	bd	bd	bd	bd	bd	bd	
	Lymphocytes	bd	bd	bd	bd	bd	bd	bd	
	Macrophages	bd	bd	bd	bd	bd	bd	bd	
	Eosinophils	bd	bd	bd	bd	bd	bd	bd	
	**Horse 3**	**1st field**	**2nd field**	**3rd field**	**4th field**	**5th field**	**Mean**	**%**	**Mucus**
Cytospin	Neutrophils	137	145	140	155	149	145	63	2
	Mast cells	0	0	0	0	0	0	0	
	Lymphocytes	37	40	35	32	38	36	15	
	Macrophages	44	51	50	48	55	50	22	
	Eosinophils	0	0	0	0	0	0	0	
Smear	Neutrophils	145	132	150	140	156	145	69	3
	Mast cells	0	0	0	0	0	0	0	
	Lymphocytes	56	42	40	32	24	39	19	
	Macrophages	27	20	30	17	29	25	12	
	Eosinophils	0	1	0	0	0	0.2	0	
	**Horse 4**	**1st field**	**2nd field**	**3rd field**	**4th field**	**5th field**	**Mean**	**%**	**Mucus**
Cytospin	Neutrophils	18	12	11	10	12	13	21	1
	Mast cells	0	0	0	0	0	0	0	
	Lymphocytes	31	32	35	32	35	33	52	
	Macrophages	22	16	15	14	18	17	27	
	Eosinophils	0	0	0	0	0	0	0	
Smear	Neutrophils	9	10	11	9	10	10	14	2
	Mast cells	0	0	0	0	0	0	0	
	Lymphocytes	43	48	49	45	44	46	66	
	Macrophages	16	14	15	13	14	14	20	
	Eosinophils	0	0	0	0	0	0	0	
	**Horse 5**	**1st field**	**2nd field**	**3rd field**	**4th field**	**5th field**	**Mean**	**%**	**Mucus**
Cytospin	Neutrophils	71	65	76	70	69	70	64	2
	Mast cells	0	0	0	0	0	0	0	
	Lymphocytes	24	22	25	22	22	23	21	
	Macrophages	17	14	16	16	17	16	15	
	Eosinophils	0	0	0	0	0	0	0	
Smear	Neutrophils	80	86	91	101	81	88	75	3
	Mast cells	0	0	0	0	0	0	0	
	Lymphocytes	20	18	18	25	19	20	17	
	Macrophages	11	7	7	16	6	9	8	
	Eosinophils	0	0	0	0	1	0.2	0	
	**Horse 6**	**1st field**	**2nd field**	**3rd field**	**4th field**	**5th field**	**Mean**	**%**	**Mucus**
Cytospin	Neutrophils	20	24	23	25	28	24	24	1
	Mast cells	0	1	0	1	0	0.4	0	
	Lymphocytes	44	40	48	44	42	44	43	
	Macrophages	32	32	30	39	34	33	33	
	Eosinophils	0	0	0	0	0	0	0	
Smear	Neutrophils	22	12	8	10	9	12	17	2
	Mast cells	0	0	0	0	0	0	0	
	Lymphocytes	22	12	16	9	10	14	20	
	Macrophages	47	38	44	43	47	44	63	
	Eosinophils	0	0	0	0	0	0	0	

Table S6. Raw data of cytocentrifugate and smear cell count results at T5.
Abbreviations: bd=below to detect

	T5								
	**Horse 1**	**1st field**	**2nd field**	**3rd field**	**4th field**	**5th field**	**Mean**	**%**	**Mucus**
Cytospin	Neutrophils	115	102	128	136	120	120	82	3
	Mast cells	0	0	0	0	0	0	0	
	Lymphocytes	20	17	19	20	21	19	13	
	Macrophages	5	7	8	7	8	7	5	
	Eosinophils	0	0	0	0	0	0	0	
Smear	Neutrophils	73	108	125	122	149	115	76	3
	Mast cells	0	0	0	0	0	0	0	
	Lymphocytes	7	25	38	33	40	29	19	
	Macrophages	7	5	9	6	13	8	5	
	Eosinophils	0	0	0	0	0	0	0	
	**Horse 2**	**1st field**	**2nd field**	**3rd field**	**4th field**	**5th field**	**Mean**	**%**	**Mucus**
Cytospin	Neutrophils	16	32	35	35	17	27	40	1
	Mast cells	0	0	0	0	0	0	0	
	Lymphocytes	22	26	28	37	28	28	42	
	Macrophages	13	11	15	11	10	12	18	
	Eosinophils	0	0	0	0	0	0	0	
Smear	Neutrophils	45	36	35	29	33	36	34	3
	Mast cells	1	0	0	0	0	0.2	0	
	Lymphocytes	73	45	40	35	42	47	45	
	Macrophages	22	27	23	26	18	23	21	
	Eosinophils	0	0	0	0	0	0	0	
	**Horse 3**	**1st field**	**2nd field**	**3rd field**	**4th field**	**5th field**	**Mean**	**%**	**Mucus**
Cytospin	Neutrophils	134	127	128	120	111	124	54	3
	Mast cells	0	0	1	1	0	0.4	0	
	Lymphocytes	62	48	32	46	31	44	19	
	Macrophages	63	65	58	60	63	62	27	
	Eosinophils	0	0	0	0	0	0	0	
Smear	Neutrophils	82	64	87	82	79	79	49	3
	Mast cells	0	0	0	0	0	0	0	
	Lymphocytes	35	41	44	25	47	38	24	
	Macrophages	50	44	38	51	35	44	27	
	Eosinophils	0	0	0	1	0	0.2	0	
	**Horse 4**	**1st field**	**2nd field**	**3rd field**	**4th field**	**5th field**	**Mean**	**%**	**Mucus**
Cytospin	Neutrophils	22	33	25	28	35	39	76	1
	Mast cells	0	0	0	0	0	0	0	
	Lymphocytes	9	5	12	5	6	7	19	
	Macrophages	3	1	4	1	2	2	5	
	Eosinophils	0	0	0	0	0	0	0	
Smear	Neutrophils	125	139	149	130	112	131	82	3
	Mast cells	0	0	0	0	0	0	0	
	Lymphocytes	11	17	15	19	19	16	10	
	Macrophages	7	12	9	15	16	12	8	
	Eosinophils	0	0	0	0	0	0	0	
	**Horse 5**	**1st field**	**2nd field**	**3rd field**	**4th field**	**5th field**	**Mean**	**%**	**Mucus**
Cytospin	Neutrophils	13	15	9	9	10	11	39	3
	Mast cells	0	0	0	0	0	0	0	
	Lymphocytes	12	10	11	10	11	11	39	
	Macrophages	7	7	6	5	7	6	21	
	Eosinophils	0	0	1	0	0	0.2	1	
Smear	Neutrophils	59	45	51	59	50	53	70	3
	Mast cells	0	0	0	0	0	0	0	
	Lymphocytes	10	8	7	8	15	10	13	
	Macrophages	12	13	17	15	10	13	17	
	Eosinophils	0	0	0	0	0	0	0	
	**Horse 6**	**1st field**	**2nd field**	**3rd field**	**4th field**	**5th field**	**Mean**	**%**	**Mucus**
Cytospin	Neutrophils	35	28	32	33	34	32	15	2
	Mast cells	0	0	0	0	0	0	0	
	Lymphocytes	99	102	95	92	100	98	46	
	Macrophages	90	82	78	79	87	83	39	
	Eosinophils	0	0	0	0	0	0	0	
Smear	Neutrophils	23	10	14	10	16	15	17	3
	Mast cells	0	0	0	0	0	0	0	
	Lymphocytes	38	30	44	51	61	45	49	
	Macrophages	24	25	35	32	37	31	34	
	Eosinophils	0	0	0	0	0	0	0	

Table S7. Raw data of cytocentrifugate and smear cell count results at T6.
Abbreviations: bd=below to detect

					T6				
	**Horse 1**	**1st field**	**2nd field**	**3rd field**	**4th field**	**5th field**	**Mean**	**%**	**Mucus**
Cytospin	Neutrophils	39	43	47	46	44	44	68	2
	Mast cells	0	1	0	1	0	0.4	1	
	Lymphocytes	14	12	11	14	12	13	20	
	Macrophages	10	6	4	6	7	7	11	
	Eosinophils	0	0	0	0	0	0	0	
Smear	Neutrophils	78	86	75	96	104	88	68	3
	Mast cells	0	0	0	0	0	0	0	
	Lymphocytes	24	33	26	37	33	31	24	
	Macrophages	10	9	11	15	9	11	8	
	Eosinophils	0	0	0	0	0	0	0	
	**Horse 2**	**1st field**	**2nd field**	**3rd field**	**4th field**	**5th field**	**Mean**	**%**	**Mucus**
Cytospin	Neutrophils	55	46	49	57	62	54	55	1
	Mast cells	0	0	1	0	0	0.2	0	
	Lymphocytes	36	25	31	32	29	31	32	
	Macrophages	8	19	12	16	11	13	13	
	Eosinophils	85	79	102	93	97	91	75	
Smear	Neutrophils	0	0	0	0	0	0	0	3
	Mast cells	18	19	12	18	13	16	13	
	Lymphocytes	27	9	12	10	11	14	12	
	Macrophages	27	9	12	10	11	14	12	
	Eosinophils	0	0	0	0	0	0	0	
	**Horse 3**	**1st field**	**2nd field**	**3rd field**	**4th field**	**5th field**	**Mean**	**%**	**Mucus**
Cytospin	Neutrophils	161	135	150	168	155	154	58	2
	Mast cells	0	0	0	0	0	0	0	
	Lymphocytes	42	33	39	49	45	42	16	
	Macrophages	75	68	65	71	67	69	26	
	Eosinophils	0	0	0	0	0	0	0	
Smear	Neutrophils	151	192	198	191	145	175	58	3
	Mast cells	0	0	0	0	0	0	0	
	Lymphocytes	37	44	35	46	40	40	13	
	Macrophages	82	95	84	102	78	88	29	
	Eosinophils	0	0	0	0	0	0	0	
	**Horse 4**	**1st field**	**2nd field**	**3rd field**	**4th field**	**5th field**	**Mean**	**%**	**Mucus**
Cytospin	Neutrophils	8	12	7	8	7	8	11	1
	Mast cells	0	0	0	0	0	0	0	
	Lymphocytes	41	46	40	45	43	43	57	
	Macrophages	24	20	23	22	24	23	32	
	Eosinophils	0	0	0	0	0	0	0	
Smear	Neutrophils	12	12	14	10	12	12	10	2
	Mast cells	0	0	0	0	0	0	0	
	Lymphocytes	63	59	60	68	69	64	54	
	Macrophages	52	44	42	40	38	43	36	
	Eosinophils	0	0	0	0	0	0	0	
	**Horse 5**	**1st field**	**2nd field**	**3rd field**	**4th field**	**5th field**	**Mean**	**%**	**Mucus**
Cytospin	Neutrophils	18	25	15	11	29	20	28	2
	Mast cells	1	2	3	2	3	2	3	
	Lymphocytes	13	23	36	40	38	30	43	
	Macrophages	25	14	24	12	15	18	26	
	Eosinophils	0	0	0	0	0	0	0	
Smear	Neutrophils	171	194	165	201	183	183	62	3
	Mast cells	0	0	0	0	0	0	0	
	Lymphocytes	79	84	77	81	89	82	28	
	Macrophages	31	29	28	33	30	30	10	
	Eosinophils	0	0	0	0	0	0	0	
	**Horse 6**	**1st field**	**2nd field**	**3rd field**	**4th field**	**5th field**	**Mean**	**%**	**Mucus**
Cytospin	Neutrophils	95	121	115	98	100	106	45	2
	Mast cells	2	0	0	0	0	0.4	0	
	Lymphocytes	69	72	60	74	98	75	32	
	Macrophages	53	56	50	42	70	54	23	
	Eosinophils	0	0	0	0	0	0	0	
Smear	Neutrophils	125	118	100	135	115	119	62	3
	Mast cells	0	0	0	0	0	0	0	
	Lymphocytes	44	42	40	50	59	47	25	
	Macrophages	33	26	18	27	20	25	13	
	Eosinophils	0	0	0	0	0	0	0	

Table S8. Raw data of cytocentrifugate and smear cell count results at T7.
Abbreviations: bd=below to detect

				T7					
	**Horse 1**	**1st field**	**2nd field**	**3rd field**	**4th field**	**5th field**	**Mean**	**%**	**Mucus**
Cytospin	Neutrophils	41	51	49	44	47	46	66	2
	Mast cells	0	1	0	0	0	0.2	0	
	Lymphocytes	11	15	15	16	14	14	20	
	Macrophages	9	10	11	10	8	10	14	
	Eosinophils	0	0	0	0	0	0	0	
Smear	Neutrophils	bd	bd	bd	bd	bd	bd	bd	bd
	Mast cells	bd	bd	bd	bd	bd	bd	bd	
	Lymphocytes	bd	bd	bd	bd	bd	bd	bd	
	Macrophages	bd	bd	bd	bd	bd	bd	bd	
	Eosinophils	bd	bd	bd	bd	bd	bd	bd	
	**Horse 2**	**1st field**	**2nd field**	**3rd field**	**4th field**	**5th field**	**Mean**	**%**	**Mucus**
Cytospin	Neutrophils	55	59	60	58	63	59	58	2
	Mast cells	0	0	0	0	0	0	0	
	Lymphocytes	29	32	35	27	28	30	30	
	Macrophages	8	12	10	18	11	12	12	
	Eosinophils	0	0	0	0	0	0	0	
Smear	Neutrophils	65	85	79	82	84	79	45	3
	Mast cells	0	0	0	0	0	0	0	
	Lymphocytes	54	47	58	49	47	51	29	
	Macrophages	52	48	43	49	42	47	26	
	Eosinophils	0	0	0	0	0	0	0	
	**Horse 3**	**1st field**	**2nd field**	**3rd field**	**4th field**	**5th field**	**Mean**	**%**	**Mucus**
Cytospin	Neutrophils	89	93	91	87	82	88	42	2
	Mast cells	0	0	0	0	0	0	0	
	Lymphocytes	61	72	69	71	75	70	33	
	Macrophages	52	57	50	48	55	52	25	
	Eosinophils	0	0	0	0	0	0	0	
Smear	Neutrophils	82	85	87	90	82	85	41	2
	Mast cells	0	0	0	0	0	0	0	
	Lymphocytes	52	60	61	50	62	57	27	
	Macrophages	70	63	74	64	58	66	32	
	Eosinophils	0	0	0	0	0	0	0	
	**Horse 4**	**1st field**	**2nd field**	**3rd field**	**4th field**	**5th field**	**Mean**	**%**	**Mucus**
Cytospin	Neutrophils	40	49	48	50	52	48	31	2
	Mast cells	0	0	0	0	0	0	0	
	Lymphocytes	66	60	55	65	67	63	40	
	Macrophages	38	42	49	49	50	46	29	
	Eosinophils	0	0	0	0	0	0	0	
Smear	Neutrophils	bd	bd	bd	bd	bd	bd	bd	Bd
	Mast cells	bd	bd	bd	bd	bd	bd	bd	
	Lymphocytes	bd	bd	bd	bd	bd	bd	bd	
	Macrophages	bd	bd	bd	bd	bd	bd	bd	
	Eosinophils	bd	bd	bd	bd	bd	bd	bd	
	**Horse 5**	**1st field**	**2nd field**	**3rd field**	**4th field**	**5th field**	**Mean**	**%**	**Mucus**
Cytospin	Neutrophils	81	75	89	91	7	83	73	3
	Mast cells	1	0	0	0	0	0.2	0	
	Lymphocytes	15	13	23	22	21	19	17	
	Macrophages	9	11	13	9	12	11	10	
	Eosinophils	0	0	0	0	0	0	0	
Smear	Neutrophils	68	85	88	94	75	82	65	3
	Mast cells	0	0	0	0	0	0	0	
	Lymphocytes	22	19	18	26	25	22	18	
	Macrophages	19	25	22	21	18	21	17	
	Eosinophils	0	0	0	0	0	0	0	
	**Horse 6**	**1st field**	**2nd field**	**3rd field**	**4th field**	**5th field**	**Mean**	**%**	**Mucus**
Cytospin	Neutrophils	22	26	33	35	32	30	28	2
	Mast cells	0	0	0	0	0	0	0	
	Lymphocytes	50	48	50	52	54	51	48	
	Macrophages	33	25	25	22	25	26	24	
	Eosinophils	0	0	0	0	0	0	0	
Smear	Neutrophils	44	47	42	48	52	47	25	2
	Mast cells	0	0	0	0	0	0	0	
	Lymphocytes	32	87	91	85	94	78	42	
	Macrophages	62	56	60	58	65	60	32	
	Eosinophils	0	0	0	0	0	0	0	
